# Economic development, urbanization, technological change and overweight: What do we learn from 244 Demographic and Health Surveys?

**DOI:** 10.1016/j.ehb.2013.11.003

**Published:** 2014-07

**Authors:** Yevgeniy Goryakin, Marc Suhrcke

**Affiliations:** a Health Economics Group, Norwich Medical School, University of East Anglia, Norwich NR4 7TJ, UK; b UKCRC Centre for Diet and Activity Research (CEDAR), Institute of Public Health, Cambridge, UK

**Keywords:** Overweight, Socioeconomic factors, Economic development, Developing countries

## Abstract

Obesity and overweight are spreading fast in developing countries, and have reached world record levels in some of them. Capturing the size, patterns and trends of the problem has, however, been severely hampered by the lack of comparable data in low and middle income countries. We seek to begin to fill this gap by testing several hypotheses on the determinants/correlates of overweight among women, related to the influence of economic and technological development. We undertake econometric analysis of nationally representative data on about 878,000 women aged 15–49 from 244 Demographic and Health Surveys (DHS) for 56 countries over the years 1991–2009. Our findings support most previously expressed hypotheses of what might explain obesity patterns in developing countries, but they also reject some prior notions and add considerable nuance to the emerging pattern.

## Highlights

• We study association between income, socioeconomic status (SES), technological change and overweight risk.
• Positive association between SES and overweight weakens as the gross domestic product (GDP) per capita goes up.
• Urban residence is positively related to overweight at all levels of GDP per capita.
• Several proxies of technological progress also positively related to overweight risk.

## 1 Introduction

Obesity has become a global phenomenon not solely confined to rich countries (James, 2008; Popkin, 2007), with the total number of overweight and obese people being estimated at about 1.5 billion in 2008 worldwide (Popkin et al., 2012). However, the accurate description of its size, trends and socioeconomic patterns has been compromised by the scarcity of globally comparable data. One recent study (Finucane et al., 2011) attempted to fill this void by estimating data on mean body mass index (BMI) among adults over the age of 20 living in 199 countries (analyzing 960 country years worth of data on 9.1 million participants). They found that between 1980 and 2008, mean BMI in all countries combined increased by 0.4 (0.5) kg/m^2^ per decade for men (women). In order to study the trends and determinants of obesity and overweight specifically in the developing countries, a small set of recent studies (Martorell et al., 2000; Mendez et al., 2005; Mendez and Popkin, 2004; Monteiro et al., 2004; Subramanian et al., 2011) has used data from the Demographic and Health Surveys (DHS), a particularly rich source of information that had hitherto primarily been used for the analysis of fertility and “traditional” developing country disease challenges in the area of maternal and child health. In this article we exploit to an even greater extent the DHS dataset, using data on almost one million women aged 15–49 from 244 Demographic and Health Surveys (DHS) for 56 countries over the years 1991–2009. We use this data to examine a broader range of questions than the previous studies that predominantly focused on how overweight, obesity or BMI in women varied between socioeconomic groups within a cross section of developing countries. By combining the DHS data with appropriate socioeconomic indicators or determinants from other sources, we are in a position to test selected hypotheses that are either derived from theoretical predictions or, for the most part, have been proposed in the literature (or expressed in the public debate) without yet having been submitted to closer empirical scrutiny.

Taken together, our findings aim to produce a set of empirically confirmed stylized facts on patterns, trends and correlates of being above normal weight (defined as having the Body Mass Index (BMI) of 25, or greater), in the developing countries. As a number of potential explanations for increasing overweight prevalence have been suggested over the last years (e.g. see Popkin et al., 2012 for a recent overview), we grouped them into two main categories (necessarily omitting some well-known potential correlates related to early life events, e.g. famine (Popkin et al., 2012), as well as genetics-related explanations): (1) those that concern the relationship between proxies of economic development and individual overweight, and (2) those examining the association between proxies of urbanization and proxies of technological change on one hand and individual overweight status on the other hand. It is important to note that we apply a somewhat more general definition of “technological change” here than in the economic growth literature, where it is more narrowly understood as a change in total factor productivity. We broadly interpret technological change as the process of invention, innovation and diffusion of various technologies (e.g. labour saving and food production ones) that may facilitate changes in energy expenditure or consumption, and therefore may affect the likelihood of being overweight or obese. There is a significant public health and economic literature which considers the term “technological change” in this similar, broader sense (e.g. Finkelstein et al., 2005; Huffman and Rizov, 2007; Lakdawalla and Philipson, 2009; Philipson, 2001; Philipson and Posner, 2003a; Swinburn et al., 2011)

Given the data constraints, we focus the empirical tests of the hypotheses on women only. We examine a third group of hypotheses, linking overweight prevalence to globalization-related determinants in a companion paper (Goryakin and Suhrcke, 2013).

### 1.1 The role of economic development in overweight

Hypothesis 1.1 Overweight is a “disease of affluence”, but only up to a point: as countries grow out of extreme poverty, overweight among women will increase. However, as countries continue to grow richer, the increase should slow down at some level of per capita income.

Why should overweight be positively related to national income? The concept of the nutrition transition (Popkin, 2001; Popkin et al., 2012) emphasizes the role played by increased affordability of processed foods, as well as foods rich in calories high in fat and sugar (both due to rising incomes, and advances in food technologies) in developing countries, especially since the 1970s. Similarly, economic theory (Philipson and Posner, 2003b) predicts that technological change (which drives and accompanies rising national income) entails lower costs of consuming calories and higher opportunity cost of expending them, which, taken together, increases weight. Within this theory, the nonlinearity can arise because greater income would also increase the demand for health (and thus a BMI closer to the medically ideal level), assuming that health is a normal good (Grossman, 1972). The theory predicts that the latter effect would at some point more than compensate the weight-enhancing effect resulting from the changes in calorie consumption and expenditure.

Hypothesis 1.2 An adverse economic shock (recession) will be associated with lower likelihood of being overweight among women.

How might body weight respond to a sudden fall in income? It is tempting to infer from the hypothesized concave and positive relationship between per capita gross domestic product (GDP) and overweight prevalence from Hypothesis 1.1 that a recession should be associated with a reduction in overweight in countries below a certain threshold of per capita GDP and either no change or even an increase in overweight in middle income countries. However, the concave relationship at one point in time across countries (which was the focus of Hypothesis 1.1) is more likely to describe a long-term association. Relationships between short-term *changes* in per capita GDP and *changes* in overweight may well differ from the long-term relationship in *levels*.

In a series of papers, Ruhm (2000, 2001, 2003, 2005, 2007) examined the impact of recession on health in a range of high income countries, finding evidence for improving (deteriorating) health in response to a recession (boom). Ruhm (2000) found that obesity declined in times of rising national unemployment rates in the US, likely due to (1) the opportunity cost of exercise falling as unemployment increases, (2) more time available for health enhancing activities, and (3) possibly due to less income being available for calorie consumption. In addition, a number of studies have shown that undernutrition prevalence increased rapidly during times of global economic crises in Cambodia, Bangladesh, Indonesia, Kenya and Mauritania (SCN, 2009). Similarly, Pongou et al. (2006) have found that child undernutrition increased considerably during times of economic crises and structural adjustment programmes in the 1990s in Cameroon. To the extent that undernutrition is the flipside of overweight, this should mean that overweight will decrease in low income countries experiencing a recession.

Hypothesis 1.3 In low income countries, women of higher socioeconomic status (SES) will be more likely to be overweight than those with lower SES, whereas in middle income countries, the burden of overweight will shift towards women of lower SES, resulting in an insignificant or mildly negative relationship between SES and the probability of being overweight.

The previous hypotheses predicted the association between national income and overweight prevalence in all population groups. By contrast, Hypothesis 1.3 considers within-country differences between women of varying socioeconomic status (SES), proxied by their level of educational attainment. The hypothesis is inspired by a widely cited study that demonstrated the shifting burden of obesity from the rich to the poor as countries’ wealth grows (Monteiro et al., 2004), using largely DHS data from 1992 to 2000 for 37 developing countries. Their results suggested that the reversal in the obesity gradient occurred at a level of GNP per capita of about 2500 US dollars, which was broadly supported by a recent systematic review (Dinsa et al., 2012). Another recent paper (Subramanian et al., 2011), that used more data from DHS, an additional SES proxy (i.e. wealth) and a more detailed analysis, found that in low income countries, the overweight burden was mostly concentrated among higher SES people, and that the wealth gradient became less marked only at a level of per capita GDP of about 5500 USD. Moreover, Neuman et al. (2011) found that the SES gradient in overweight prevalence did not weaken over time in the low-to middle income countries studied.

The theoretical justification for this hypothesis can be linked to the explanation given in the Hypothesis 1.2: before the threshold per capita income is reached, the higher SES people will have greater income, greater demand for calories and therefore greater weight. However, as economic development progresses, the demand for thinness and health (backed by better access to healthier lifestyle opportunities) among higher SES women may start to outweigh the demand for calories faster than among women belonging to lower SES groups (Jones-Smith et al., 2011), thus leading to the switchover at a certain income level.

In addition, some empirical evidence (Popkin, 2001) suggests that the income elasticity of demand for certain foods high in fat is higher for people belonging to lower SES classes. If this is indeed the case, then rising income levels among the poor may lead to greater relative consumption of unhealthy diets among them, compared to people from upper SES classes.

### 1.2 The role of urbanization and technological change in overweight

Our next set of hypotheses reflects the widespread notion that urbanization and technological change have been driving the rise in overweight and obesity. Previous studies have linked urbanization with various technologies potentially associated either with the reduction in energy expenditure over time (Monda et al., 2007; Popkin, 1999; Rivera et al., 2002; Swinburn et al., 2011), or with more abundant supply and consumption of cheaper, energy dense foods (Drewnowski and Popkin, 1997; Popkin, 1999; Popkin and Gordon-Larsen, 2004). Therefore, we have subsumed several hypotheses concerning the role of urbanization and different manifestations of technological change under the same group of hypotheses.

Hypothesis 2.1 Living in urban areas will be associated with higher probability of being overweight among women.

The strong positive association between urbanization and obesity has been examined and confirmed quite extensively in previous research (e.g. Martorell et al., 2000; Mendez et al., 2005; Mendez and Popkin, 2004; Subramanian et al., 2011), although Neuman et al.’s recent paper (2013) suggests that the association between urban residence and excess BMI may be mediated by higher SES of urban residents. The novelty of our approach is in the use of a much larger set of individual-level data collected in 56 countries, and in the non-parametric approach used to examine the effect of living in urban areas on overweight, by taking into account the level of economic development across countries.

In general, living in urban areas can increase the odds of being overweight for a number of reasons. For example, people living in cities may expend less energy than those living in rural areas, possibly because they have fewer opportunities to exercise, because they prefer to spend more time doing leisure activities (such as TV watching) at home, or because they work in less physically demanding occupations. In addition, those who live in the cities may transition faster to the so-called “Western” diets rich in calories from fats and refined carbohydrates, but relatively poor in calories from fruit, vegetables and cereals (Popkin, 2001; Popkin et al., 2012). This may occur for a variety of reasons, including greater exposure to food processing technologies, as well as marketing and distribution systems that promote fast and processed foods consumption. Next, we consider specific ways in which urbanization can affect the likelihood of being overweight:

Hypothesis 2.2 Possession of assets that facilitate sedentary behaviour will be associated with greater overweight prevalence among women

Technological change has given rise to an environment that may make it harder (and thus more costly) to spend calories, with cars and TVs being but two very widespread manifestations thereof. We hypothesize that owning a car or a TV set will be related to greater risk of being overweight. The explanation is intuitive: having a car may predispose people to walk less frequently, and therefore spend less energy (Sassi, 2010). Previous research has demonstrated that owning a motorized vehicle was strongly correlated with the probability of being obese in China (Bell et al., 2002). Similarly, people who own a TV set are expected to be less likely to exercise and, hence, less likely to “spend” their calories. They may also consume more energy-dense food while watching TV (Roemling and Qaim, 2012). We will separately also consider if there is a relationship between frequency of TV viewing and overweight risk. TV viewing may expose people to mass advertising of certain foods that may encourage people to snack excessively and may thereby contribute to weight gain (Finkelstein et al., 2005). Some empirical work in this area (mostly confined to higher income countries) has largely focused on individual measures of exposure to TV (e.g. number of hours per day watching TV, measured by self-completed diaries, e.g. (Cutler et al., 2003), or possession of a TV (Roemling and Qaim, 2012)). Overall, this evidence suggests a rather limited role played by TV exposure, at least in the high income countries studied (Finkelstein et al., 2005), although Roemling and Qaim (2012) did find that owning a TV was related to a small but significant increase in BMI in Indonesia. Note that our goal here is to try and isolate the overweight-enhancing influence of owning a car and a TV set, rather than the effect of socioeconomic status and income, which can also be correlated with owning a TV. Our dataset will allow us to control for both per capita national income as well as for individual socioeconomic status.

In addition, technological change and urbanization are often hypothesized to lead to increased supply and, hence, consumption of higher energy and fat density foods (Popkin, 1999, 2006, 2007). Our data will allow us to test the following related hypothesis:

Hypothesis 2.3 A greater per capita supply of calories will be associated with higher overweight probability among women.

Some research has argued that an increase in the consumption of calories played a greater role in the rise in obesity, compared to a reduction in energy expenditure (Cutler et al., 2003; Finkelstein et al., 2005). Similarly, proponents of the nutrition transition theory emphasize the role played by the growing proportion of calories obtained from diets higher in fats and refined carbohydrates, and not so much the increased calories per se (Popkin, 2001). A recent prominent study (Swinburn et al., 2011) differentiated roughly between two groups of global determinants of obesity and overweight: those related to the changes in the built environment, and those related to changes in the food supply. The authors argued that the built environment has not changed simultaneously and universally to lead to observed changes in people's weight around the world; hence, the more important drivers are attributed to the food system domain (including greater supply of cheap, energy-dense foods, as well as more advanced distribution and marketing systems to increase people's access to these foods). Similarly, Cutler et al. (2003) argued that although per capita energy expenditure in the US decreased from 1.69 and 1.57 kcal/min/kg between 1965 and 1975, it remained fairly steady during the period of fast growing obesity prevalence. On the other hand, available information on trends in energy intakes in the US suggests that calorie consumption was significantly higher in 1990s compared to the early 1980s (Finkelstein et al., 2005).

Although changes in food systems encompass much more than changes in per capita supply of calories (one reason being that the available calorie data is imperfect, and food supply does not accurately capture the calorie consumption by people living in these countries), testing if there is country-level association between per capita calorie availability and probability of being overweight, using data from 56 low and middle income countries, may still be very instructive, as the available evidence so far has been scarce, and largely confined to high income countries. On a macro level, limited empirical evidence exists that increased food energy supply was positively correlated with greater weight in USA and UK (Swinburn et al., 2011). On a micro level, a systematic review of several experimental as well as large prospective cohort studies concluded that there is strong evidence on the link between fast food consumption and increasing weight (Rosenheck, 2008). One study which examined the association between caloric intake and weight in a middle income country (Russia) concluded that the relationship was strong and positive (Huffman and Rizov, 2007). By contrast, comparing trends in obesity and diet patterns in the US population, other research found a counter-intuitive coincidence of decreasing calorie consumption and growing obesity, which was tentatively attributed to even greater reductions in energy expenditures resulting from physical inactivity (Heini and Weinsier, 1997).

Technological change has also entailed a shift in the employment of people from agriculture and industry to the service sector, which in turn may have led to the reduction in energy expenditure:

Hypothesis 2.4 Working in the service sector is associated with a greater likelihood of being overweight among women.

This may be the case because working in the service industry is – on average – less physically taxing than manual labour in agriculture. However, the existing empirical evidence is far from unambiguous. Thus, as Finkelstein et al. (2005) noted, it is true that in the US, for example, the proportion of workers employed in the manufacturing industries fell from 27% in 1980 to 19% in 2000, and that the proportion of obese men has almost doubled over this period. However, the proportion employed in manufacturing was considerably higher in 1960, i.e. 35%, and yet the proportion of men who were obese changed little between 1960 and 1980. Hence, a more fine-grained analysis, using individual-level data, is needed. We will test this hypothesis with individual-level indicators on the occupational status of women contained in the DHS dataset.

## 2 Methods

### 2.1 Data

The data in this study comes from several sources: the Demographic and Health Surveys (DHS), World Development Indicators (WDI),^1^ the Cross-National Time-Series Data Archive (CNTS)^2^ and FAOSTAT.^3^ The main source is the DHS: nationally representative, population based surveys, which collect extensive information in the areas of population, health and nutrition. DHS surveys are extensively described elsewhere (Corsi et al., 2012b; Subramanian et al., 2011). The WDI, CNTS and FAOSTAT datasets provide a range of relevant country-level economic indicators that we employed for our hypothesis-testing. The individual-level DHS data used in this paper covers 19 years (1991–2009) over 56 countries. In the regression analysis, all country-level variables were taken from WDI, CNTS and FAOSTAT datasets, and merged with individual-level DHS data for respective countries and years. More information on the data sources used is provided in Appendix.

As the full data was only available on women aged 15–49, we necessarily had to restrict our analysis to this group. The main outcome variable of interest is being above normal weight (or overweight, for short), defined as BMI greater or equal to 25 kg/m^2^. In order to trim outliers, observations for women whose BMI exceeded 50, or were less than 13, or whose weight was either greater than or equal to 220 kg, or less than or equal to 20 kg, were dropped. In addition, observations for women whose height was recorded as either greater than or equal to 2.2 m, or less than or equal to 0.9 m, were also dropped. We restricted the sample to women who were non-pregnant at the time of the interview (about 7.9% of surveyed women aged 15–49 were pregnant). In all countries, information was collected on women of child-bearing age, and thus included those who had never had children. The proportion of women with no children (which included women with and without pregnancies at the time of the interview) varied from about 7–8% of the sample (e.g. in Egypt) to about 45% (e.g. in Morocco), and this difference was largely due to the age structure of the surveyed population. Thus, in Egypt, only about 8% of surveyed women were under the age of 20 in 1995, while in Morocco in 2003, about 28% of surveyed women were under 20. This issue may be important for the comparison of overweight prevalence rates across countries, but we take care of it by adjusting estimates by sampling weights provided with the DHS surveys.

Overall, in the pooled sample, data on BMI was available for about 72% of the full sample of 1,225,808 non-pregnant women. Again, there was some variation across countries in terms of the proportion of non-pregnant women with available BMI data (from 99% in Uzbekistan, to only 26% in Brazil), that we account for when estimating country-specific overweight prevalence by employing sampling weights.

In our analysis we used education as the main socioeconomic variable of interest. While socioeconomic status can also be proxied by other variables – e.g. wealth, as was done by Subramanian et al. (2011) – we preferred education, because the way it is defined in the DHS set (using six educational quartiles, ranging from incomplete primary school to higher education), it may be considered reasonably comparable across countries as a proxy for the *absolute* level of socioeconomic status. By contrast, wealth – as it is commonly constructed on the basis of DHS data – is a *relative* measure of SES specific for each country, and thus is harder to compare when all countries with different income levels are pooled together. Nevertheless, we also tested the robustness of our findings to the inclusion of an alternative wealth measure (i.e. car ownership) as education may not fully capture the income and wealth-related aspects of socioeconomic status.

Education was defined using DHS dummies for six levels: people with no, incomplete primary, complete primary, incomplete secondary, complete secondary, and higher education. For the graphical analysis (e.g. Fig. 4), we divided education into four levels: (1) no education, (2) incomplete primary and complete primary, (3) incomplete secondary and (4) complete secondary and higher education. We then compared levels 1 and 4. Note that this gradation is different from the one used in some earlier papers (e.g. Monteiro et al., 2004), which defined top and bottom educational quartiles by the number of years of schooling in each country. We decided not to follow their approach in order to avoid dealing with countries for which too many observations were clustered around a certain value (e.g. zero years of education). In such cases, it may not be possible to clearly define four separate quartiles, and one would only be able to deal with three (or even two) groups (Monteiro et al., 2004). In our approach, the rule for selecting top and bottom “quartile” will apply equally to all countries.

A country was defined as being in economic recession if the country's GDP per capita (in purchasing power parities [PPP], constant international $, 2005) decreased by 1% or more between adjacent years. We also checked the robustness of the results by defining a shock as a more severe economic contraction, i.e. a 5% reduction in GDP per capita year-on-year. Information on the working status for a woman was based on self-reports, and we captured service sector employment using an occupational status dummy. Specifically, occupations were first aggregated into three groups as follows: (1) unemployed; (2) services (professional and managerial; clerical; sales; household and domestic; services); (3) agriculture (agriculture employed and self-employed); (4) manual (skilled manual; unskilled manual). Furthermore, for the services group a dummy was assigned with a value of one, while for manual and agriculture occupations the assigned value was zero. The availability of cars and TV sets was measured by individual-level indicators on the ownership of these items. The hypothesis on the supply of calories was tested using country-level data on average food supply (kcal per capita per day) derived from FAO balance sheets. Table A1 in the Appendix describes all the variables used in this paper.

In the regression analysis, we were interested in the association between several potential correlates and the composite category of “being above normal weight”. We therefore assigned a value of one to people whose BMI was greater than or equal to 25, and a value of zero to people whose BMI was less than 25. We also considered including the continuous variable BMI as the outcome variable, as was done in a recent paper by Subramanian et al. (2011). However, a change in BMI may have very different implications, depending on the initial value in its distribution. For example, the change in BMI from 18 to 19 (i.e. from being malnourished to having normal weight – a desirable outcome) has very different implication compared to an increase in BMI from 24 to 25 (i.e. from having normal weight to being overweight – an undesirable outcome). Measuring the association between covariates and BMI would not capture this difference, while measuring the effect of covariates on overweight (treated as a dummy variable) does.

Most hypotheses require either splitting the sample, or introducing an interaction term to distinguish between low- and middle-income countries. In this analysis, we used the World Bank year-specific thresholds defining the split between low income and middle income countries. We then either split the sample between these two groups and ran the regression analyses separately, or we introduced an interaction term between the World Bank income level and the main independent variable of interest.

### 2.2 Econometric specifications

Several econometric specifications were tested in this paper. First, to test Hypotheses 1.1, 1.3 and 2.1, we estimated the parameters of the following specification, splitting the sample into (1) low and (2) middle income countries:

(1) $$Y_{cit}=\beta X_{cit}+\delta C_{ct}+D_{t}+\alpha_{c}+e_{cit}$$

where *Y*_*cit*_ is a dummy for being above normal weight (including obese) for individual *i* living in country *c* at time *t*; *X*_*cit*_ is a set of individual-level independent variables that vary over time, which will include both the main variables of interest (e.g. education), as well as controls (e.g. age); *C*_*ct*_ is a set of country level variables that can vary over time; *D*_*t*_ is a set of time dummies that will control for trend effects, and *e*_*cit*_ is an error term. Our interest lies in estimating parameters *β* and *δ*. For correct estimation of standard errors and appropriate test statistics, the errors will be clustered at community level.

The estimates also allow controlling for time-invariant country-specific effects *α*_*c*_. For example, a country may have a certain endowment of human or natural resources that contributes to changes in the prevalence of being overweight, as well as to variation in some of the observed determinants included in the model. Not controlling for such factors may contribute to a bias in the estimated parameters. Note that this model will allow parameter estimation only when the main variables of interest are either individual-level ones or are country-level variables that vary over time. In addition, including time dummies *D*_*t*_ will allow us to control for other potential time dependence or for any world-wide factors (e.g. global economic crises) that could affect our associations of interest.

The remaining hypotheses (1.2, 2.2, 2.3, 2.4) will be tested using the following specifications:

(2.1) $$Y_{cit}=\beta X_{cit}+\delta M_{ct}+\gamma (X_{cit}*M_{ct})+D_{t}+\alpha_{c}+e_{cit}$$

(2.2) $$Y_{cit}=\beta C_{ct}+\delta M_{ct}+\gamma (C_{ct}*M_{ct})+D_{t}+\alpha_{c}+e_{cit}$$

where *M*_*ct*_ is a dummy for living in a middle income country. Interacting this variable with the main variables of interest contained in vector *X*_*cit*_ in model (2.1), and with variables contained in vector *C*_*ct*_ in model (2.2) will allow us to test if there is any differential effect of these variables between poor and richer countries. Model 2.2 will apply whenever our main variables of interest (e.g. recessions and per capita calorie consumption) vary at country level, and model 2.1 will apply when they vary at individual level (e.g. car or TV ownership). Again, we will also include the country effects *α*_*c*_ as a robustness check for each model.

As we are interested not only in the estimation of the main parameters of interest, but also in whether they are statistically significantly different in low and middle income countries, ideally we would have preferred to use specifications (2.1) or (2.2) only. However, as we have six separate educational categories, and our goal was to take advantage of the whole educational distribution, interacting each educational category separately with the medium income dummy would not produce very intuitively interpretable results. Therefore, we split the sample to estimate the association of education with overweight separately in low and medium income countries, as per specification (1). As we also had to control for urban residence and GDP per capita in such models anyway, we presented the results for these three hypotheses using the same specification (1).

## 3 Results

**Fig. 1:**
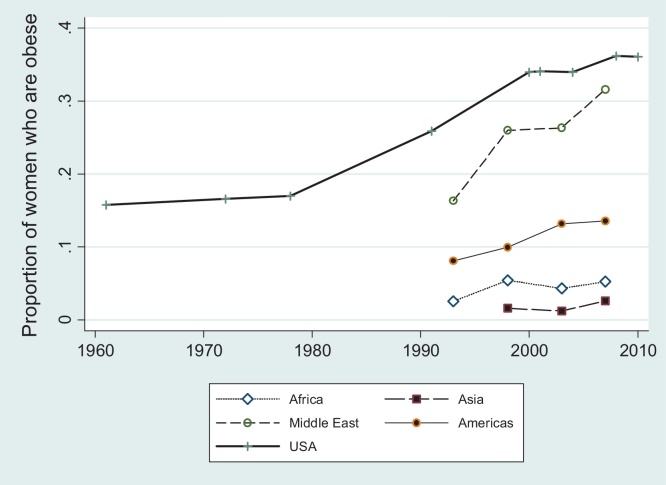
Prevalence of being obese for different regions at different stages of development, 1960–2010.

Source: DHS and NHANES datasets. Country-specific prevalence estimated using DHS survey weights. Each point represents a region-specific prevalence of female obesity, averaged over countries belonging to the respective regions during the chosen time period, and using each country's population as a weight. The following time intervals were chosen for DHS countries: 1991–1995; 1996–2000; 2001–2005; 2006–2009. In USA, the following intervals were used: 1960–1962; 1971–1974; 1976–1980; 1988–1994; 2009–2010.

In order to put the obesity “epidemic” in developing countries into perspective and evaluate its comparative severity, it may be informative to start by comparing what developing countries have recently been experiencing with what today's high income countries had experienced at earlier stages of the obesity epidemic. Although some recent studies have looked at the long-term trends in obesity and overweight prevalence in the US (e.g. Finkelstein et al., 2005; Flegal et al., 1998), there was so far no attempt to compare these trends with those observed in low and middle income countries from different regions. Fig. 1 compares the trends in the prevalence of female obesity between a prominent high-income country (USA) and the four regions that we have defined. The US data for women was taken from Flegal et al. (1998) and CDC (2010), while the prevalence data for the developing countries was estimated from DHS data. Please note that in this one instance, we consider the prevalence of being obese rather than above normal weight, to ensure comparability between the DHS data with that from the US. Specifically, for each multi-year period, we constructed a region-specific prevalence of the indicator of interest, averaging over countries belonging to the respective regions, and using each country's population as a weight. We describe the regional split in more detail in the Appendix, Table A2.

Although the trends presented in Fig. 1 do not represent a formal econometric test and therefore are only indicative, they do paint an interesting picture: the Middle East appears as by far the most worrying region in terms of obesity trends. Back in 1960, the USA already had reached a considerably higher GDP per capita than the Middle East has in 2009, and yet it still lagged behind the Middle East for most of the observed period in terms of female obesity prevalence. Hence one may conclude that the obesity challenge faced by this region does substantially exceed what even the US has had to confront. Given that the Middle East still has a lower per capita income than the US did in the 1960s, further economic development is likely to pose considerable public health challenges in this region. The comparison to the other regions is more problematic in that they are at even lower per capita incomes than the Middle East countries on average and thus the US obesity data would have to go back even further in time to allow for a meaningful comparison.

### 3.1 The role of economic development in overweight

The results of our first set of more formally tested hypotheses – the role of economic development in overweight – are reported below:

Hypothesis 1.1 Overweight is a disease of affluence – up to a point? (cross-country perspective)

**Fig. 2:**
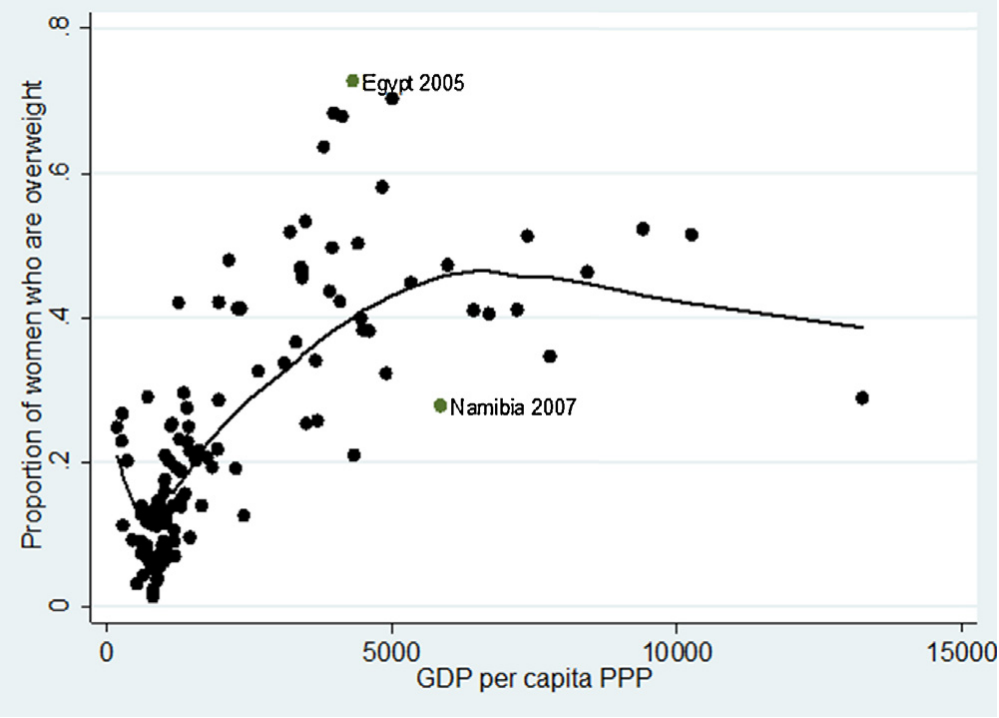
Lowess curve for the prevalence of overweight by GDP per capita, 1991–2009.

Source: DHS dataset, 1991–2009. Each dot represents a country and year specific prevalence of being above normal weight among women. Country-specific prevalence estimated using DHS survey weights. The observations for all years are pooled together.

A simple graphical illustration can be used as an initial exploration of Hypothesis 1.1. In Fig. 2, we fitted a lowess plot for the relationship between the country-level proportion of women above normal weight (i.e. having BMI greater or equal than 25), and GDP per capita, measured using PPP. This very simple analysis suggests that, indeed, the association between these two variables of interest is positive and stronger in low-income countries than in the middle-income ones. As Fig. 2 shows a very simple relationship between the level of economic development and overweight prevalence, without controlling for any socioeconomic or political factors (likely to vary widely between countries), this finding should not be over-interpreted. Nevertheless, the observed pattern confirms earlier findings derived from smaller samples of countries and individuals (Monteiro et al., 2004; Martorell et al., 2000; Ezzati et al., 2005; Egger et al., 2012).

A few interesting cases are worth highlighting: in 2007 Egypt had a far greater female overweight prevalence than could be predicted from their per capita GDP level. On the other hand, another African country – Namibia – was in the reverse situation, being located below the regression line. The high prevalence of obesity in Egypt relative to its GDP level was discussed in other studies (e.g. Martorell et al., 2000). Future research should try to identify the factors that help account for these large differentials.

**Fig. 3:**
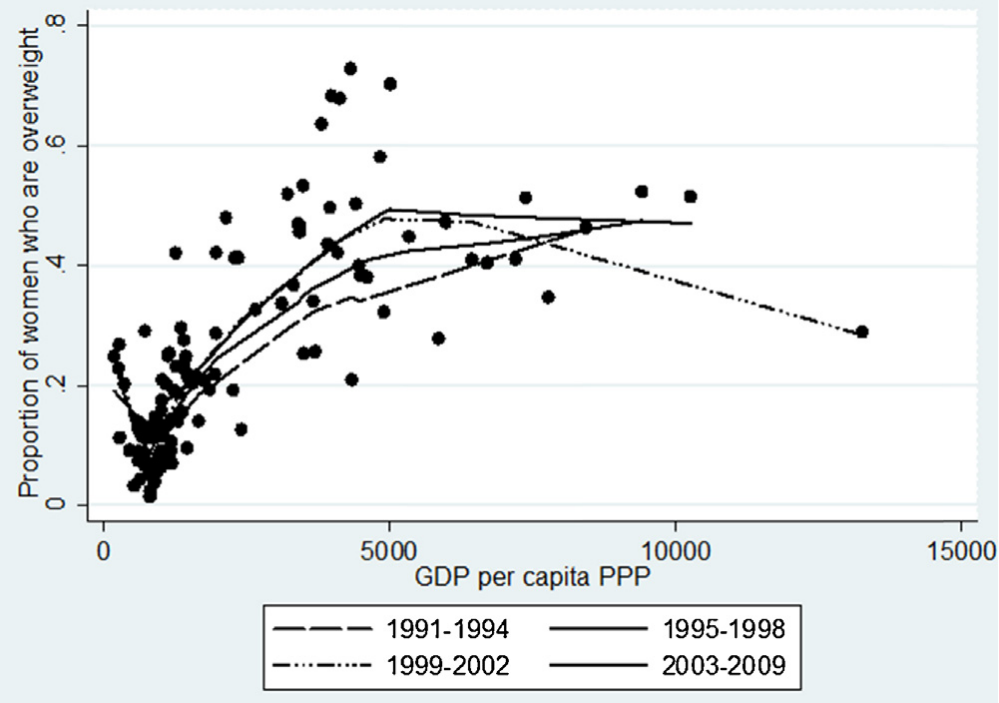
Lowess curve for the prevalence of overweight by GDP per capita and by different time periods, 1991–2009.

Source: DHS dataset, 1991–2009. Each dot represents a country and year specific prevalence of being above normal weight among women. Country-specific prevalence estimated using DHS survey weights. The observations for all years are pooled together.

In Fig. 3, we examine how the same relationship may have evolved over time. It emerges that the peak at which the prevalence of being overweight is reached is at approximately USD 5000–6000 per capita, PPP. Moreover, it appears that this peak level has been slightly shifting to the left over time, suggesting the existence of a learning process, which allows countries that reach higher income levels to switch to a descending trajectory for overweight in the following time periods.

On the other hand, it is quite worrying that for a given level of economic development, the prevalence of overweight tends to increase over time. Thus, at USD 5000 GDP per capita, the prevalence of being above normal weight among women in 1991–1994 was approximately 35%. In 2003–2009, this proportion increased to almost 50%. A similar upward shift of the income-BMI curve over time was observed in another study, using US data (Ezzati et al., 2005). This patterning emphasizes once again the role played by determinants of overweight other than national income, the influence of which was apparently increasing over time. It is also worth noting that this reflects the reverse picture of Preston Curve (Preston, 1975) which indicates an improvement in life expectancy over the decades (attributed to technological change), while in the case of overweight we observe a deterioration. Given the trends mentioned in a previous paragraph, it appears that as time passes, countries may learn to switch to a downward trajectory faster (i.e. at lower level of GDP), but before doing so they tend to reach a greater overweight prevalence than previously.

A more formal test of this hypothesis can be performed with the help of simple regression analysis as outlined in equation 1 above, using individual-level overweight status as an outcome variable. In Table 1, column 1, we see that the log of GDP per capita generally has a positive association with the probability of being overweight, suggesting that a 10% increase in GDP per capita is associated with about 1.2 percentage points (p.p.) higher probability of a woman living in that country being overweight. Put differently, a twofold increase in a country's GDP from, say, 500 to 1000 USD, is predicted to lead to about 12 p.p. higher probability of being overweight. This finding is robust to including country-level fixed effects as outlined in column 2 (although the magnitude of the effect becomes smaller). This is a relatively small effect, but note that it is averaged over all regions and years, and is derived after controlling for educational attainment and for the proportion of people living in urban areas.

By contrast, results in columns 3 and 4 (as well as in 5 and 6) show that the estimate of the association between log GDP and the probability of being overweight among women is insignificant for the sample of low income countries, and positive and significant for middle income countries. This finding contrasts with the picture presented in Fig. 2. We will come back to possible explanation for this finding in the Additional Checks section below.

Hypothesis 1.2 An adverse economic shock (recession) will be associated with a lower likelihood of being overweight among women.

The results for Hypothesis 1.2 are summarized in columns 1 and 2 of Table 2. The first column provides the OLS results. We see that economic decline, as measured by a dummy for countries that experienced a decline of 1% or more in their real per capita GDP compared to the previous year, is associated with an increase in the probability of being overweight in low income countries, and a decrease in the likelihood of being overweight in middle income countries. This finding is, however, not robust to including country-level fixed effects (see column 2): the fixed effects results show that with economic decline people in poor countries are less likely to be overweight – as Hypothesis 1.2 predicted – while those living in richer countries gain weight. Note that when we defined an economic shock differently, i.e. as a contraction by 5% or more in real GDP per capita, we obtained very similar results in the model without country fixed effects (CFE), and somewhat different results for the model with CFE. Specifically, while the interaction parameter was very similar, the economic shock parameter for low income countries was now positive and significant (b = 0.03), suggesting that the effect of a more significant economic shock may operate differently in the lower-income countries. Nevertheless, as only 3% of the DHS sample lived during years of such strong recessions, it is important to be cautious in interpreting this specific finding.

We can also test more directly how work status of women is correlated with being overweight. In Table 2, columns 7 and 8 we see that in low income countries, working is surprisingly negatively correlated with overweight, while the reverse is true in middle income countries.

Our findings thus partly support the previous evidence of a positive association between country-level recessions and weight reduction in the low income countries (SCN, 2009). As for the middle income countries, our findings contradict our hypothesis (and thus the work by Ruhm (2000, 2001, 2003, 2005, 2007)) but are in line with the small strand of literature arguing that recessions increase the propensity to be overweight (Morris et al., 1992; Ludwig and Pollack, 2009). One potential explanation for this finding is that in the more developed countries, economic recessions (and loss of income from the main breadwinner) may lead to greater employment of women to supplement family income as a coping mechanism (Lim, 2000). In turn, they may have less time for cooking and may thus be more likely to be overweight. On the other hand, the reverse may be true for women living in the less developed countries, who may have to cut their total consumption of calories altogether (thereby losing weight). Nevertheless, there is reason to doubt this explanation in that the scope of finding work during recessions may be quite limited for the previously unemployed women. Instead, it is possible that the observed increase in overweight among women in recession-hit middle income countries, and the reverse trend in the low income countries, is due to different patterns of dietary response. For example, women in poor countries may consume fewer calories during recessions due to more binding constraints on overall food-related spending, while women in middle-income countries may have an option to switch to less healthy, high calorie food instead. Unfortunately, it is not possible to explore this issue further given the available data. Having said that, the finding that *individual* working status is positively associated with overweight and obesity in the middle income countries, is in line with the literature that argues that being employed may be related to fewer opportunities for exercise and taking care of one's health, as well as to a line of reasoning that women who work may be less likely to spend time cooking nutritious food, thus contributing to the increase in overweight (Finkelstein et al., 2005).

Hypothesis 1.3 In low income countries, women of higher socioeconomic status (SES) will be more likely to be overweight than those with lower SES, whereas in middle income countries, the burden of overweight will shift towards women of lower SES, resulting in an insignificant or mildly negative relationship between SES and the probability of being overweight.

The association between education and the probability of being overweight is generally positive for the overall sample (Table 1, columns 1 and 2). Thus, women who have the least education, are about 11p.p. less likely to be overweight than those with the most education. The country fixed effects specification gives very similar results. Again, this number conceals variation between low and middle income countries: columns 3 and 4, as well as 5 and 6 in Table 1, show that at all levels of education, the association between this measure of socioeconomic status and the probability of being overweight is weaker for the sample of middle income countries. Moreover, when country-level fixed effects are used, the relationship between education and probability of being overweight actually turns negative in middle-income countries, as expected. This finding was affected to only a small extent by the inclusion of the alternative wealth measure, i.e. car ownership (results not shown here but available from the authors upon request).

**Fig. A1:**
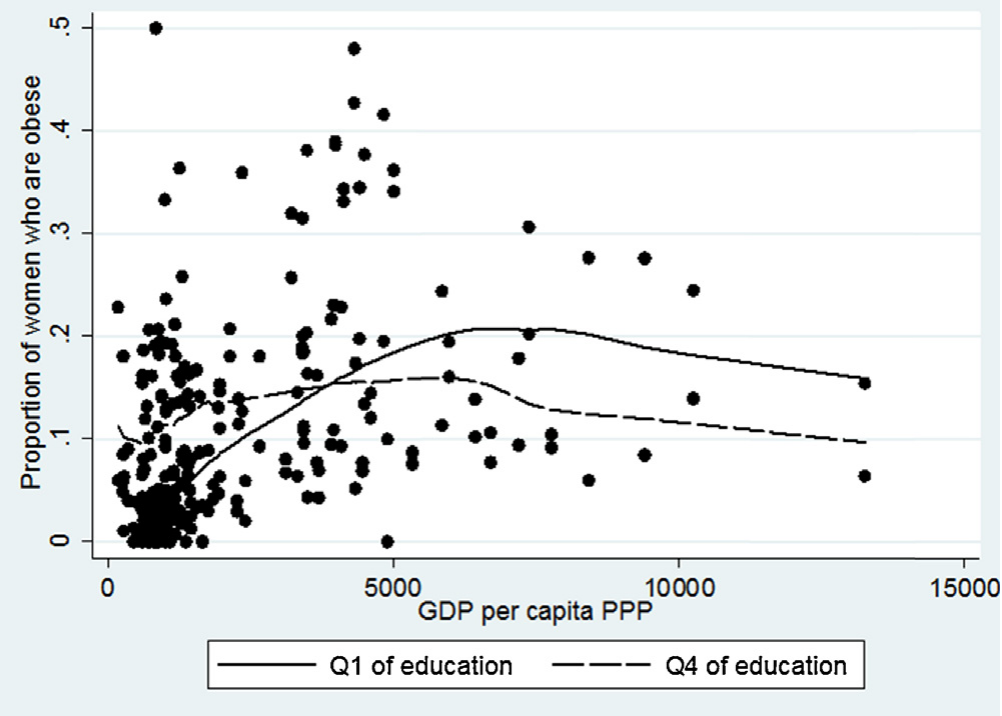
Lowess curve for the prevalence of obesity by education, 1991.

Source: DHS dataset, 1991–2009. Each dot represents a country and year specific prevalence of being obese, for a respective educational group. Country-specific prevalence estimated using DHS survey weights. Q1 refers to women with no education; Q4 – to women with complete secondary and higher education.

**Fig. 4:**
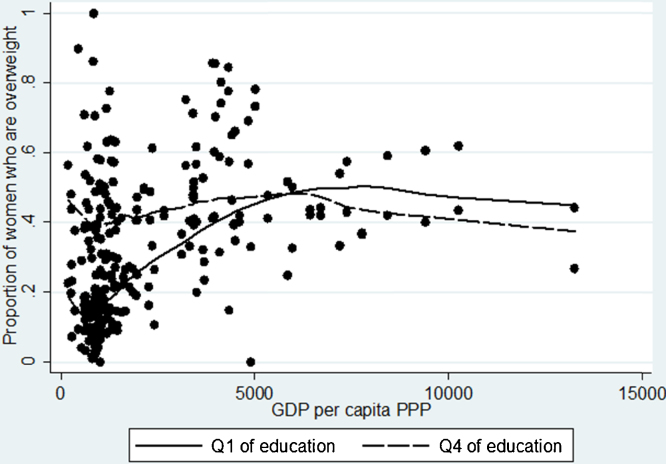
Lowess curve for the prevalence of overweight by education, 1991–2009.

Source: DHS dataset, 1991–2009. Each dot represents a country and year specific prevalence of being overweight, for a respective educational group. Country-specific prevalence estimated using DHS survey weights. Q1 refers to women with no education; Q4 – to women with complete secondary and higher education.

Additional evidence on Hypothesis 1.3 is also presented in Fig. 4 below, showing that up until about 5000–6000 USD GDP per capita (PPP), those in the top education quartile are more likely to be overweight than those in the bottom quartile. Beyond this threshold, however, it is those with the least education who have consistently greater probability of being overweight. This association becomes even more pronounced – and the threshold declines to around 4000 USD – when the obesity prevalence is measured on the vertical axis (see Fig. A1 in the Appendix).

Note that our finding is consistent with the results of other, smaller-scale studies. For example, a negative association between education and obesity probability was found for women living in China (Xuhong et al., 2008), Brazil (Marins et al., 2007), Eastern Europe (Pikhart et al., 2007), Iran (Hajian-Tilaki and Heidari, 2009), Argentina (Fleischer et al., 2008) – all countries at a level of around 4000 GDP per capita (PPP, international 2005 $) or higher. In contrast, many earlier studies focussing on less developed countries tended to find positive associations between education and obesity (Sobal and Stunkard, 1989). In a recent study on the relationship between educational attainment and overweight among Chinese women (Jones-Smith et al., 2011), no relationship was found using 1989 data, but a strongly negative relationship (OR = 0.22 (CI 0.11–0.42)) was found for 2006. In another recent study that used World Health Survey data (Moore et al., 2010), the general finding was a positive relationship between education and overweight. Note, however, that their results were not split by country income classification, so it is hard to directly compare the results to ours.

Monteiro et al. (2004) also found that as national income increased the burden of obesity was shifting from the top to the bottom socioeconomic status. The fact that our finding is similar, using considerably more data, and using a different definition for the top and bottom educational attainment, is encouraging. Similarly, Martorell et al. (2000) found that more educated women living in very poor countries were more likely to be obese than the least educated ones, while this gradient disappeared as countries got richer. On the other hand, Subramanian et al. (2011) used wealth as an additional proxy for socioeconomic status. Their finding was different in that there was no shift in the burden of obesity from the richest to the poorest people as national income was increasing (although there was a shift when the richest quartile was compared to the third and second quartile). That said, those results are not directly comparable to ours, since they used predicted BMI level, rather than prevalence of being above normal weight as an outcome variable. They also found that the association between wealth and BMI was considerably stronger than the association between education and BMI. However, since including both education and wealth in the same equation may mean that the influence of one variable is captured in another, it is hard to conclude from this with certainty that wealth is more important than education for nutritional health.

### 3.2 The role of urbanization and technological change in overweight

In what follows we summarize the results from our four hypotheses that could be subsumed under the factors capturing indicators of technological change.

Hypothesis 2.1 Living in urban areas will be associated with higher probability of being overweight among women.

**Fig. 5:**
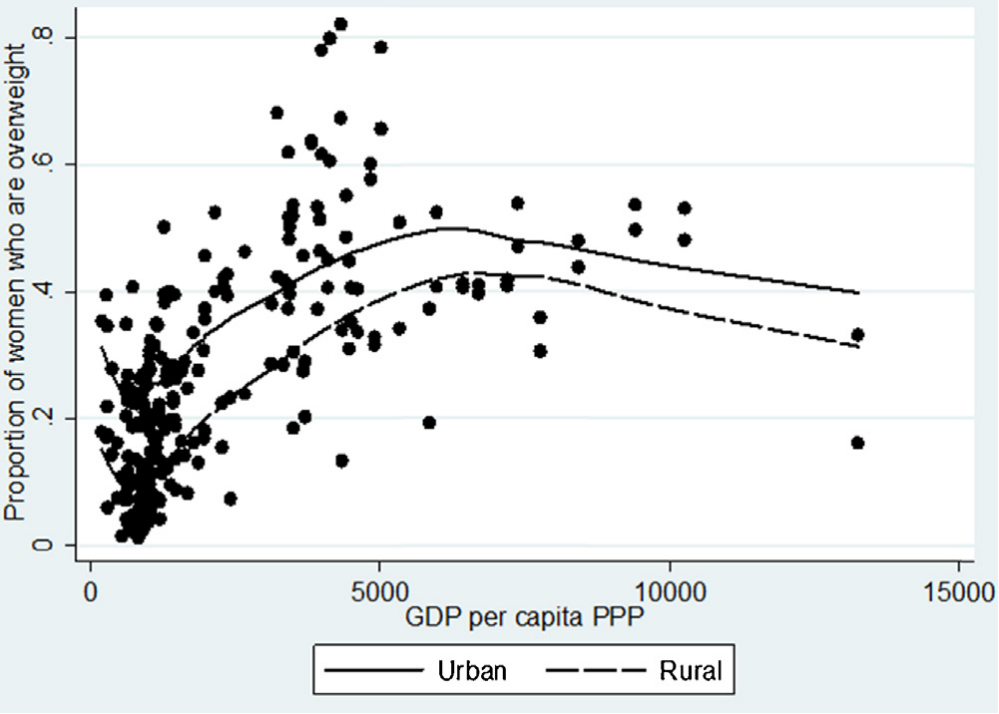
Lowess curve for the prevalence of being overweight by residence, 1991–2009.

Source: DHS dataset, 1991–2009. Each dot represents a country and year specific prevalence of being overweight, for a respective residence group. Country-specific prevalence estimated using DHS survey weights.

The tests of Hypothesis 2.1 are also contained in Table 1. It is obvious that this hypothesis is supported by the results from all specifications, suggesting that, in general, living in urban areas is related to an about 7–12 p.p. greater probability of being overweight, even after controlling for education. Moreover, the effect of urban residence on overweight is weaker in countries with a higher level of income – consistent with the finding from another study (Popkin et al., 2012) which used a different method, and different data – again suggesting a potential learning curve that occurs in countries as they undergo economic transformation. Also, Fig. 5 shows that for all levels of GDP per capita, the probability of being overweight is higher in urban areas, and thus unlike education, there appears to be no switchover in obesity occurring from urban to rural areas as national income grows larger. This confirms previous findings by Mendez et al. (2005), which however where based on less comprehensive data and used a smoothed plot, rather than lowess regression. Similarly, a recent paper using data from 42 low and middle income countries (Popkin et al., 2012) also found that on average, urban women had higher baseline prevalence of being overweight, compared to rural women, and this was true for all countries they studied. Moreover, they found that in countries with higher GDP per capita, there was relatively little association between residence and overweight prevalence, and that this association was much stronger in lower income countries. Given the worldwide trend towards urbanization that is set to continue in developing countries (United Nations, 2011), the consistently overweight-enhancing effect of urbanization does raise concerns that this will remain a factor driving a further spread of obesity in the developing world.

Hypothesis 2.2 Possession of assets that facilitate sedentary behaviour will be associated with greater overweight prevalence among women.

Hypothesis 2.2 was confirmed both in OLS specification and CFE specifications (see Table 2). Thus, having one car is associated with an about 12–14 p.p. higher probability of being overweight. The effect was also considerably stronger in the low-income countries compared to the middle income ones, suggesting that changing transportation habits there may entail a particularly high risk of adverse weight outcomes. Similarly, TV ownership was significantly related to female overweight in both OLS and fixed effects specification (see Table 2). Interestingly, the magnitude of association is very similar to what we found for car ownership variable, suggesting that having a TV is related to an about 12 p.p. greater probability of being overweight among women in the low income countries. The association becomes slightly smaller in size in the middle income countries when country fixed effects are included. We also found a quite strong dose-response relationship between frequency of watching TV and overweight risk (using the country fixed effects specification – detailed results available upon request). Thus, compared to the reference group of never watching TV, those who watched it less than once a week; at least once a week; and almost every day, had an 4.5 p.p., 7.2 p.p. and 11 p.p. greater risk of being overweight, respectively.

Hypothesis 2.3 A greater per capita supply of calories will be associated with higher overweight probability among women.

The food supply hypothesis was tested by running specifications reported in columns 9–10 of Table 2. As expected, there is a significant positive association between average, country-level per capita supply of calories (for convenience of interpretation, expressed in 1000 kcal per capita) and the probability of female overweight in the simple OLS model. The effect is also stronger in the middle income countries. To put this into perspective, the average total supply of calories, across all countries, is about 2400 kcal/person/day. Increasing this availability by 1000 kcal, or by about 41%, would lead to about 11.9 p.p. greater probability of women being overweight in the middle income countries. The effect remains significant for the middle income countries in both models. It should be emphasized that this is a test of the relationship between average per capita supply of calories and the probability of being overweight, and, as noted above, the former is a crude measure of the actual consumption of calories in the country. This measure does not take into account the potential changes in the patterns of the diet either, e.g. the potential shift to higher consumption of calories from refined carbohydrates and fats (Popkin et al., 2012). Nevertheless, the size of the association that we found for the middle income countries supports the hypothesis that greater supply of food plays a role in increasing overweight risk, at least in the middle income countries.

Hypothesis 2.4 Working in the service sector is associated with a greater likelihood of being overweight among women.

This hypothesis was also confirmed in both the OLS and fixed effects specification (see columns 3 and 4 in Table 2). Thus, working in the service sector was associated with an about 8 p.p. higher probability of being overweight among women, and the effect was somewhat weaker in the middle income countries (although it still remains significant).

## 4 Additional checks

**Fig. 6:**
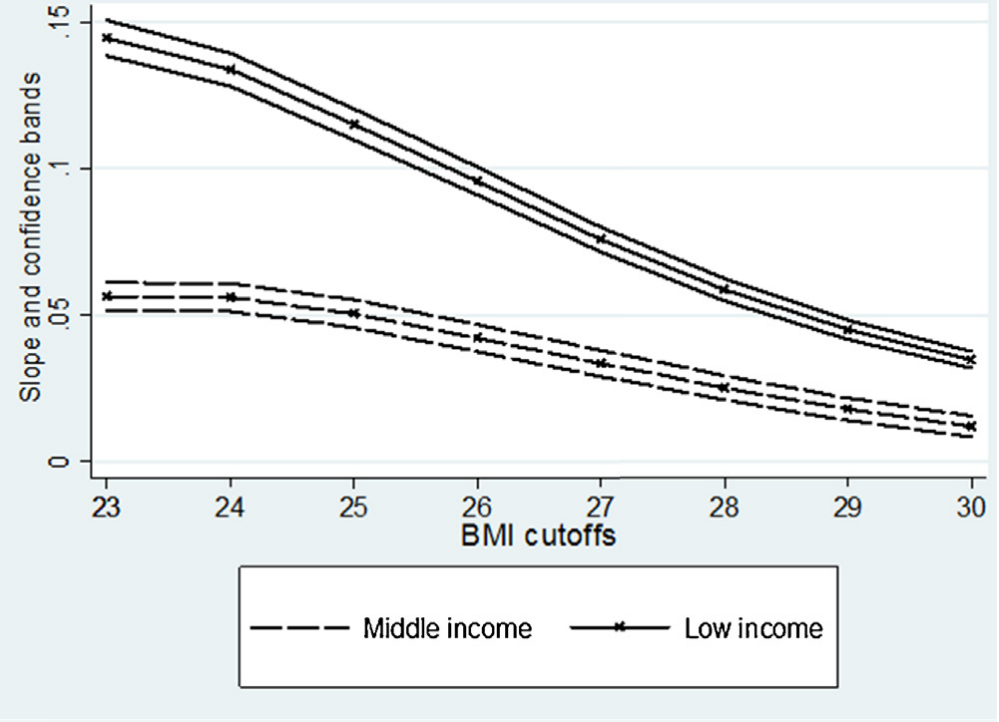
Association between complete university education and being above normal weight.

Source: DHS dataset, 1991–2009. On vertical axis, parameter values from linear probability regression of overweight on university education, with 95% confidence intervals, are reported. On horizontal axis, values of this parameter for alternative BMI cut-offs defining being above normal weight are given. All models contain the same control variables as those presented in Table 1.

One potential concern with our findings is that traditionally used BMI cut-off points ultimately are arbitrary and at least in some countries or regions inappropriate to capture the prevalence of weight problems (James, 2004, 2008). To tackle this problem, we ran the same regressions, but with differently defined cut-offs for being overweight. The results are presented in Fig. 6, focussing – for illustrative purposes – on the role of education in overweight and its sensitivity to changes in the cut-off. All underlying regression estimates included the same controls as in Table 1. In the low-income countries, the effect of education is very sensitive to the cut-offs used. Thus, when being overweight is defined as having a BMI greater or equal to 23, the people with some or complete university education have about 15 p.p. greater probability of being overweight than the reference group. However, this differential falls by more than 50, to about 9% p.p., when the cut-off is at about 26. The effect of university education on being overweight and obese is at its smallest when the BMI cut-off is 30. Comparing this picture to the one emerging for middle income countries (Fig. 6), we can see that there is much less sensitivity in the effect of education by different BMI cut-offs.

We also checked how sensitive the results were to using different thresholds defining the split between low and middle income countries.

Table 3 shows that the OLS results were particularly sensitive for the log GDP per capita variable (please note that OLS version was chosen over country fixed effects, as there was an insufficient number of countries to estimate parameters for log GDP per capita variables in columns 2 and 4).

It is important to bear in mind that in the results presented in Table 1, the effect of GDP on the probability of being overweight was surprisingly insignificant in low income countries and positive in middle income ones. However, now we see that this is only true when comparatively low income levels are chosen for the threshold separating low from middle income countries. When the middle income level was defined as having a GDP per capita greater than 3000 USD or higher (PPP, 2005 international dollars), our earlier unexpected finding is reversed, and is now in line with the original hypothesis. Note that this finding is also in line with what we found in Fig. 2. Education turned out as another category that is quite sensitive to the cut-offs used: the relationship in the predicted direction was again at its strongest when a higher cut-off was used. There was little change in the association between urban living and being overweight regardless of the threshold level chosen. Table 4 provides the same information for the remaining hypotheses (for convenience, only the main parameter estimates are presented). We see that in this case the choice of the cut-off had little effect on almost all variables. Hence we conclude that the choice of a cut-off for the income level made a substantial difference only for Hypotheses 1.1 and 1.2. Note that in this case again the OLS version was chosen over country fixed effects, as there was an insufficient number of countries to estimate several parameters in columns 4 and 5.

We also examine whether the effect estimates of the main determinants vary by region. In Table 5 we present OLS results for the equation reported in the first two columns of Table 1, but this time split by 4 regions, and for two different outcomes: being above normal weight (i.e. when BMI is 25 or greater), and being obese (i.e. when BMI is 30 or greater). Here the OLS version was again chosen instead of country fixed effects estimates, as there was an insufficient number of countries to estimate parameters on the log GDP per capita variable for columns 4 and 8. Several interesting findings emerge. First, in the Americas and the Middle East, education has a different association with the probability of being overweight among women, than in Africa and Asia (qualitatively, the same results are obtained when using obesity as the outcome variable.) While in the Americas and the Middle East, women with the most education have a lower probability of being overweight than almost all other educational categories (except the lowest one), this is not the case in other regions, where less education is generally associated with a lower probability of being overweight. A possible explanation for this pattern is that most countries in the Americas and Middle East are already past the GDP threshold at which the overweight burden tends to shift to lower socioeconomic groups.

In addition, log GDP per capita has a considerably stronger association with overweight in the Middle East: in this region a 10% greater GDP is associated with an about 12 p.p. higher probability of being overweight. It appears that in the Middle East, economic development may fuel the rise in overweight prevalence much faster than in the rest of the world. Also, the association between age and obesity is much stronger in the Middle East and in the Americas than in other regions (not shown here). This implies that as the population ages in those regions, the obesity burden there may become particularly acute.

## 5 Conclusions

While the growing obesity challenge in the developing world may have been fairly widely acknowledged by now, precisely capturing the size, patterns, trends and determinants of overweight and obesity in developing countries has thus far been severely hampered by the lack of comparable micro data in low and middle income countries. To date, most of the broadly related studies focussed either on calculating country-specific prevalence of obesity, or on examining how socioeconomic status of individuals and their place of residence is related to obesity burden. By taking advantage of a considerably bigger sample than used in previous studies, we have re-examined previous findings and have tested a number of additional hypotheses derived from existing theoretical literature, or from widely held notions about alleged determinants of being overweight, that had been taken for granted without having been empirically tested. Wherever possible, we tested for several hypotheses simultaneously, by including relevant variables of interest at the same time.

On the whole, our results confirmed the set of hypotheses about the association between economic development and overweight:

(1) Hypothesis 1.1: The relationship between national per capita income and obesity is positive and concave;
(2) Hypothesis 1.2: In an economic recession, people in poor countries lose weight and, hence, are less likely to be overweight (although this was not the case in the middle income countries);
(3) Hypothesis 1.3: The relationship between education (as a proxy for socioeconomic status) and the probability of being overweight is positive in the low income countries and negative in medium-income countries.

The latter finding in particular suggests an important policy implication: in order to minimize negative consequences of economic development in the form of greater overweight, there may be reason to target attention at preventing overweight and obesity among higher socioeconomic groups in low income countries, and at lower socioeconomic groups – in middle income countries. Moreover, it appears that the greatest returns to better nutritional status from more education are in the Americas and the Middle East regions (see Table 5). Given that the Middle East is the most problematic region in terms of obesity prevalence, this finding is especially promising, as additional investment into female education in this region is likely to bring the greatest returns in terms of reducing overweight.

The picture emerging from the next set of hypotheses suggests that urbanization and related technological change do play at least some role in the growing overweight prevalence:

(1) Hypothesis 2.1: Our finding that living in urban areas is related to significantly higher likelihood of being overweight for countries at all income levels suggests that the continuous urbanization process taking place in the developing countries poses a serious public health challenge.
(2) Hypothesis 2.2: Both car and TV ownership are robustly related to overweight outcomes, with the magnitude of the association being smaller (yet still positive) in the middle income countries compared to the low income countries.
(3) Hypothesis 2.3: Greater per capita calorie intake is positively related to overweight, but only in the middle income countries.
(4) Hypothesis 2.4: Shifting patterns of employment from agriculture into services, traditionally associated with urbanization and technological change are also significantly related to a greater probability of being overweight in both low and middle income countries.

In addition, we have dealt with the previously mentioned cut-off problem (e.g. James, 2004, 2008) in a systematic way. For example, we have demonstrated that the results can be quite sensitive to choosing different thresholds for defining overweight. Moreover, we checked how the estimation results varied depending on the level of income threshold chosen to differentiate between low and middle income countries.

Inevitably, our study suffers from several limitations. For example, the sample was necessarily restricted to women only, mostly of child-bearing age. Therefore, generalizing our findings to women of all age groups, let alone to both genders, is impossible. Nevertheless, since the age group 15–49 represents the most economically active group of women, who also typically have a number of people depending on them (including children and the elderly), focussing our attention on this demographic segment seems to cover a sizeable and important share of the population.

Also, by the very nature of the sampling undertaken for the DHS surveys, most sampled women were mothers with at least one child under 5 years of age (Monteiro et al., 2004). This can be problematic in that such women may be more likely to be overweight, and therefore the estimated prevalence of obesity may be slightly overestimated. Nevertheless, since our goal was not to estimate country-level obesity prevalence, but rather to test the statistical association between overweight status and its correlates, this should not be a significant concern in our study.

The sample composition was also changing depending on the availability of certain variables. For example, information on the occupational status of respondents was available for about 50% of the sample with BMI information, while for most other variables the data was much more available. In addition, since the data we used is of a cross-sectional nature, we have to caution against deriving too strong causal inferences about the identified relationships. Rather, our goal was a more modest test for any potential partial correlation between a number of relevant socioeconomic variables and overweight. Finally, we did not explore how variation in overweight status was partitioned between national and community levels. A recent paper by Corsi et al. (2012a), using similar data, did focus on this aspect.

Despite the limitations, this study presents an important step towards quantitatively examining the role that different proxies of economic development, urbanization and technological change play in explaining overweight in developing countries, using a unique global dataset. While the findings support several previously expressed hypotheses of what might explain obesity patterns in developing countries, they also reject some prior notions, and add considerable nuance to the emerging pattern. More research is needed to assess the extent to which the findings reflect a true causal relationship.

## Figures and Tables

**Table A1:** Variable definitions.

Variable name	Type	Source	Additional definition
Overweight	Individual	DHS	Defined as dummy = 1 if BMI ≥25
No education	Individual	DHS	N/A
Incomplete primary education	Individual	DHS	N/A
Complete primary education	Individual	DHS	N/A
Complete secondary education	Individual	DHS	N/A
Complete secondary education	Individual	DHS	N/A
Higher education	Individual	DHS	N/A
Urban	Individual	DHS	Urban residence
Age	Individual	DHS	Split into 3 categories: 15–24, 25–34, 35–49 years
Working	Individual	DHS	Respond currently working
Number of children	Individual	DHS	Split into four categories: 0, 1–2, 3–5, 6 and more children
Service occupation	Individual	DHS	Working in professional and managerial, clerical, sales, household and domestic, services occupations
Passenger cars	Individual	DHS	N/A
TV set	Individual	DHS	N/A
Log GDP per capita	Country	WDI	Log GDP per capita, PPP (constant 2005 international $)
Recession	Country	WDI	Difference between current and lagged for 1 year values of GDP per capita, PPP (constant 2005 international $), is negative
Total calories per capita	Country	FAOSTAT	Food supply (kcal/capita/day)

**Table A2:** Regional split.

**Asia**: Bangladesh; Cambodia; India; Kazakhstan; Kyrgyzstan; Nepal; Philippines; Uzbekistan
**Africa**: Burkina Faso; Benin; Central African Republic; Democratic Republic of the Congo; Cameroon; Chad; Comoros; Congo Brazzaville; Cote d’Ivoire; Ethiopia; Gabon; Ghana; Guinea; Kenya; Lesotho; Liberia; Madagascar; Malawi; Mali; Mozambique; Namibia; Niger; Nigeria; Rwanda; Senegal; Sierra Leone; South Africa; Swaziland; Tanzania; Togo; Zambia; Zimbabwe
**Americas**: Bolivia; Brazil; Colombia; Dominican; Guatemala; Haiti; Honduras; Nicaragua; Peru
**Middle East**: Egypt; Jordan; Morocco; Turkey

Note that there were too few countries in the European category (Armenia, Azerbaijan, Moldova), therefore we decided to exclude them from the subgroup analysis.

**Table 1:** Effect of covariates on overweight risk. Testing Hypotheses 1.1, 1.3 and 2.1.

	Overweight	Overweight	Overweight	Overweight	Overweight	Overweight
	(1)	(2)	(3)	(4)	(5)	(6)
			Low	Medium	Low	Medium
No education	−0.112***	−0.111***	−0.179***	−0.042***	−0.166***	−0.075***
	(0.003)	(0.003)	(0.004)	(0.004)	(0.004)	(0.004)
Incomplete primary	−0.039***	−0.039***	−0.115***	−0.011***	−0.114***	0.010***
	(0.003)	(0.003)	(0.004)	(0.004)	(0.004)	(0.004)
Complete primary	−0.019***	−0.017***	−0.081***	−0.005	−0.090***	0.027***
	(0.003)	(0.003)	(0.004)	(0.004)	(0.004)	(0.004)
Incomplete secondary	−0.025***	−0.011***	−0.072***	−0.019***	−0.066***	0.018***
	(0.003)	(0.002)	(0.004)	(0.003)	(0.004)	(0.003)
Complete secondary	0.038***	0.007***	−0.003	0.054***	−0.029***	0.034***
	(0.003)	(0.003)	(0.004)	(0.003)	(0.004)	(0.003)
University	*Reference*	*Reference*	*Reference*	*Reference*	*Reference*	*Reference*
Urban	0.104***	0.105***	0.116***	0.073***	0.112***	0.089***
	(0.002)	(0.001)	(0.002)	(0.003)	(0.002)	(0.002)
Rural	*Reference*	*Reference*	*Reference*	*Reference*	*Reference*	*Reference*
Log GDP per capita	0.121***	0.088***	0.002	0.109***	0.017	1.006***
	(0.001)	(0.010)	(0.002)	(0.004)	(0.012)	(0.068)
15–24 years	−0.233***	−0.232***	−0.189***	−0.310***	−0.188***	−0.295***
	(0.002)	(0.002)	(0.002)	(0.003)	(0.002)	(0.003)
25–34 years	−0.124***	−0.122***	−0.103***	−0.150***	−0.100***	−0.144***
	(0.001)	(0.001)	(0.002)	(0.002)	(0.002)	(0.002)
35–49 years	*Reference*	*Reference*	*Reference*	*Reference*	*Reference*	*Reference*
0 children	−0.062***	−0.038***	−0.013***	−0.125***	−0.015***	−0.100***
	(0.002)	(0.002)	(0.002)	(0.004)	(0.002)	(0.004)
1–2 children	0.021***	0.034***	0.039***	0.007**	0.044***	0.004
	(0.002)	(0.002)	(0.002)	(0.003)	(0.002)	(0.003)
3–5 children	0.042***	0.045***	0.035***	0.062***	0.038***	0.044***
	(0.002)	(0.001)	(0.002)	(0.003)	(0.002)	(0.003)
6 and more children	*Reference*	*Reference*	*Reference*	*Reference*	*Reference*	*Reference*

Observations	878,310	878,310	529,416	337,837	529,416	337,837
*R*-squared	0.185	0.094	0.115	0.150	0.093	0.114
Country FE	No	Yes	No	No	Yes	Yes

Notes: Cluster-robust standard errors in parentheses (based on DHS cluster identifiers). All specifications are OLS models or country fixed effects models. *p < 0.1.

** *p* < 0.05.

*** *p* < 0.01. All specifications contain time dummies.

**Table 2:** Effect of covariates on overweight risk. Testing Hypotheses 1.2, 2.2–2.4.

	(1)	(2)	(3)	(4)	(5)	(6)
	Overweight	Overweight	Overweight	Overweight	Overweight	Overweight
Recession	0.054***	−0.010**				
	(0.003)	(0.005)				
Recession*MI	−0.154***	0.076***				
	(0.006)	(0.010)				
Service			0.090***	0.082***		
			(0.002)	(0.002)		
Service*MI			−0.047***	−0.030***		
			(0.004)	(0.003)		
Cars					0.136***	0.120***
					(0.003)	(0.003)
Cars*MI					−0.105***	−0.081***
					(0.004)	(0.004)

Observations	855,380	855,380	460,435	460,435	791,385	791,385
*R*-squared	0.200	0.095	0.196	0.084	0.199	0.096
Country FE	No	Yes	No	Yes	No	Yes

	(7)	(8)	(9)	(10)	(11)	(12)
Working	−0.022***	−0.012***				
	(0.001)	(0.001)				
Working*MI	−0.022***	0.042***				
	(0.002)	(0.002)				
Total kcal (1000), pc			0.026***	−0.015		
			(0.004)	(0.015)		
Total kcal (1000), pc*MI			0.198***	0.119***		
			(0.006)	(0.019)		
TV sets					0.125***	0.115***
					(0.002)	(0.002)
TV sets*MI					0.055***	−0.027***
					(0.003)	(0.003)

Observations	865,846	865,846	771,178	771,178	844,385	844,385
*R*-squared	0.199	0.094	0.209	0.094	0.214	0.103
Country FE	No	Yes	No	Yes	No	Yes

Notes: Cluster-robust standard errors in parentheses (based on DHS cluster identifiers). All specifications are OLS models or country fixed effects models. *p < 0.1.

** *p* < 0.05.

*** *p* < 0.01. All specifications contain time dummies, a dummy for middle income country category, as well as the same control variables as in Table 1. “pc” means per capita; “MI” refers to middle income country indicator. Total calories, recession and MI are country-level covariates, the rest are individual level.

**Table 3:** Effect of covariates on overweight risk. Robustness checks for alternative GDP per capita cut-offs separating low from middle-income counties.

	(1)	(2)	(3)	(4)	(5)	(6)	(7)	(8)	(9)	(10)
	<5000	≥5000	<4000	≥4000	<3000	≥3000	<2000	≥2000	<1000	≥1000
No education	−0.121***	−0.047***	−0.136***	−0.025***	−0.172***	−0.018***	−0.202***	−0.086***	−0.148***	−0.098***
	(0.003)	(0.007)	(0.003)	(0.006)	(0.003)	(0.005)	(0.005)	(0.004)	(0.009)	(0.003)
Incomplete primary	−0.046***	0.004	−0.052***	−0.003	−0.109***	−0.021***	−0.140***	−0.024***	−0.106***	−0.031***
	(0.003)	(0.006)	(0.003)	(0.005)	(0.003)	(0.004)	(0.005)	(0.003)	(0.009)	(0.003)
Complete primary	−0.022***	0.033***	−0.032***	0.032***	−0.076***	0.019***	−0.104***	0.011***	−0.082***	−0.017***
	(0.003)	(0.006)	(0.003)	(0.005)	(0.004)	(0.004)	(0.005)	(0.004)	(0.009)	(0.003)
Incomplete secondary	−0.030***	0.009*	−0.036***	0.004	−0.059***	−0.008**	−0.089***	−0.019***	−0.078***	−0.025***
	(0.003)	(0.005)	(0.003)	(0.004)	(0.003)	(0.004)	(0.005)	(0.003)	(0.009)	(0.003)
Complete secondary	0.030***	0.018***	0.023***	0.023***	0.001	0.059***	−0.040***	0.041***	−0.026***	0.033***
	(0.003)	(0.005)	(0.003)	(0.004)	(0.004)	(0.004)	(0.005)	(0.003)	(0.010)	(0.003)
University	*Reference*	*Reference*	*Reference*	*Reference*	*Reference*	*Reference*	*Reference*	*Reference*	*Reference*	*Reference*
Urban	0.114***	0.072***	0.122***	0.071***	0.115***	0.067***	0.119***	0.068***	0.116***	0.095***
	(0.002)	(0.004)	(0.002)	(0.003)	(0.002)	(0.003)	(0.002)	(0.003)	(0.003)	(0.002)
Rural	*Reference*	*Reference*	*Reference*	*Reference*	*Reference*	*Reference*	*Reference*	*Reference*	*Reference*	*Reference*
Log GDP per capita	0.142***	−0.187***	0.114***	−0.349***	0.017***	−0.124***	0.011***	0.114***	−0.085***	0.164***
	(0.002)	(0.015)	(0.002)	(0.009)	(0.002)	(0.007)	(0.002)	(0.005)	(0.003)	(0.002)

Observations	776,751	101,559	715,317	162,993	598,520	279,790	450,395	427,915	206,832	671,478
*R*-squared	0.184	0.180	0.154	0.195	0.116	0.153	0.100	0.191	0.085	0.186

Notes: Cluster-robust standard errors in parentheses (based on DHS cluster identifiers). All specifications are OLS models. Note that OLS version was chosen over country fixed effects, as there was insufficient number of countries to estimate parameters for log GDP per capita variables in columns 2 and 4 in CFE version. Each column refers to alternative cut-off chosen to define split between low and middle income countries. In odd columns, parameters for alternative definitions of low income countries are presented. In even columns, parameters for alternative definitions of middle income countries are presented.

* *p* < 0.1. **p < 005.

*** *p* < 0.01. All specifications contain time dummies, as well as age/children number categories.

**Table 4:** Robustness checks for alternative GDP per capita cut-offs for defining middle-income counties (Hypotheses 1.2, 2.2–2.4), OLS models.

	(1)	(2)	(3)	(4)	(5)
	≥1000	≥2000	≥3000	≥4000	≥5000
Recession	0.098***	0.051***	0.041***	0.046***	0.049***
	(0.005)	(0.003)	(0.003)	(0.004)	(0.004)
Recession_INT_	−0.159***	−0.149***	−0.172***	−0.230***	−0.162***
	(0.006)	(0.007)	(0.007)	(0.008)	(0.010)
Service	0.085***	0.087***	0.086***	0.093***	0.091***
	(0.003)	(0.002)	(0.002)	(0.002)	(0.002)
Service_INT_	−0.005	−0.006*	−0.067***	−0.109***	−0.109***
	(0.003)	(0.003)	(0.004)	(0.005)	(0.006)
Cars	0.161***	0.135***	0.138***	0.121***	0.098***
	(0.007)	(0.004)	(0.003)	(0.003)	(0.002)
Cars_INT_	−0.086***	−0.074***	−0.129***	−0.118***	−0.083***
	(0.007)	(0.005)	(0.004)	(0.005)	(0.006)
Working	−0.035***	−0.018***	−0.022***	−0.031***	−0.031***
	(0.002)	(0.001)	(0.001)	(0.001)	(0.001)
Working_INT_	0.002	−0.014***	−0.012***	−0.013***	−0.007**
	(0.002)	(0.002)	(0.003)	(0.003)	(0.004)
Total kcal (1000), pc	0.001	0.043***	0.076***	0. 174***	0. 207***
	(0.006)	(0.004)	(0.039)	(0.004)	(0.003)
Total kcal (1000), pc_INT_	0.21***	0.223***	0.135***	0.083***	−0.064***
	(0.007)	(0.006)	(0.005)	(0.006)	(0.008)
TV sets	0.145***	0.135***	0.138***	0.149***	0.150***
	(0.004)	(0.002)	(0.002)	(0.002)	(0.002)
TV sets_INT_	0.006	0.017***	0.011***	0.007	−0.006
	(0.004)	(0.003)	(0.003)	(0.004)	(0.005)

Notes: Cluster-robust standard errors in parentheses (based on DHS cluster identifiers). All specifications are OLS models. Note that OLS version was chosen over country fixed effects, as there was insufficient number of countries to estimate several parameters in columns 4 and 5 in CFE version.

* *p* < 0.1. **p < 0.05.

*** *p* < 0.01. All specifications contain time dummies, as well as the same control variables as in Table 1. Each column presents results for alternative cut-offs of middle income countries. Each row represents main parameter only, for different levels of GDP per capita (purchasing power parity, constant 2005 dollars) reflected in columns. The INT subscript refers to the interaction between the main parameter of interest and the dummy for the respective income threshold level. Total calories and recession are country-level covariates, the rest are individual level. For example, consider the effect of working on the probability of being above normal weight, for the case when middle income countries are defined as having GDP per capita, PPP of 2000 dollars or more. The results are presented in column 2. For women living in low income countries, being employed reduces the probability of being above normal weight by about 1.8 p.p. In the middle income countries, the effect of being employed is also negative, and is (statistically significantly) stronger than in the low income countries, by about 1.5 p.p.

**Table 5:** Correlates of being above normal weight/obese, by region.

	(1)	(2)	(3)	(4)	(5)	(6)	(7)	(8)
	Overweight	Overweight	Overweight	Overweight	Obese	Obese	Obese	Obese
	ME	Americas	Africa	Asia	ME	Americas	Africa	Asia
No education	−0.066***	−0.049***	−0.235***	−0.162***	−0.008	−0.011***	−0.099***	−0.039***
	(0.006)	(0.006)	(0.006)	(0.005)	(0.007)	(0.004)	(0.004)	(0.002)
Incomplete primary	−0.005	0.042***	−0.167***	−0.118***	0.055***	0.039***	−0.071***	−0.028***
	(0.007)	(0.005)	(0.006)	(0.005)	(0.007)	(0.003)	(0.004)	(0.002)
Complete primary	0.029***	0.065***	−0.129***	−0.085***	0.071***	0.050***	−0.062***	−0.017***
	(0.008)	(0.005)	(0.006)	(0.005)	(0.008)	(0.003)	(0.004)	(0.002)
Incomplete secondary	0.002	0.044***	−0.112***	−0.057***	0.042***	0.039***	−0.051***	−0.009***
	(0.006)	(0.004)	(0.006)	(0.004)	(0.007)	(0.003)	(0.004)	(0.002)
Complete secondary	0.020***	0.037***	−0.053***	−0.012**	0.038***	0.023***	−0.030***	0.005^*^
	(0.006)	(0.005)	(0.006)	(0.005)	(0.006)	(0.003)	(0.004)	(0.003)
University	*Reference*	*Reference*	*Reference*	*Reference*	*Reference*	*Reference*	*Reference*	*Reference*
Log GDP capita	1.234***	0.092***	0.033***	0.053***	0.735***	0.024***	0.020***	0.029***
	(0.039)	(0.005)	(0.002)	(0.004)	(0.032)	(0.003)	(0.001)	(0.002)
Urban	0.099***	0.094***	0.113***	0.092***	0.090***	0.059***	0.043***	0.028***
	(0.004)	(0.003)	(0.002)	(0.003)	(0.004)	(0.002)	(0.001)	(0.001)
Rural	*Reference*	*Reference*	*Reference*	*Reference*	*Reference*	*Reference*	*Reference*	*Reference*

Observations	100,108	172,812	377,032	191,904	100,108	172,818	377,032	191,904
*R*-squared	0.197	0.125	0.108	0.137	0.147	0.071	0.065	0.054

Notes: Cluster-robust standard errors in parentheses (based on DHS cluster identifiers). All specifications are OLS models. Note that OLS version was chosen over country fixed effects, as there was insufficient number of countries to estimate parameters on log GDP per capita variable for columns 4 and 8 in CFE version. *p < 0.1.

** *p* < 0.05.

*** *p* < 0.01. All specifications contain time dummies, as well as age/children number categories.

## Footnotes

1 http://data.worldbank.org/data-catalog/world-development-indicators.

2 http://www.databanksinternational.com.

3 faostat.fao.org.
